# Cysteine Dioxygenase Enzyme Activity and Gene Expression in the Dimorphic Pathogenic Fungus *Histoplasma capsulatum* Is in both the Mold and Yeast Morphotypes and Exhibits Substantial Strain Variation

**DOI:** 10.3390/jof6010024

**Published:** 2020-02-13

**Authors:** Melissa A. Adams, Glenmore Shearer

**Affiliations:** Center for Molecular and Cellular Biosciences, The University of Southern Mississippi, 118 College Dr. #5018, Hattiesburg, MS 39406, USA; Melissa.Adams@colin.edu

**Keywords:** cysteine dioxygenase, cysteine oxidase, *CDO1*, *Histoplasma* dimorphism, mold, yeast

## Abstract

In the dimorphism (mold/yeast) *Histoplasma* *capsulatum* (*Hc*) literature are reports that yeast (the so-called pathogenic form) uniquely expresses a cysteine dioxygenase (CDO, approx. 10,500 dal) activity which the mold morphotype (the so-called saprophytic soil form) does not express (C.F., Kumar et al., Biochem 22, 762, 1983). This yeast-specific CDO activity is postulated to play a critical role in the mold-to-yeast shift. A number of years ago, our lab isolated the gene encoding the *Hc* cysteine dioxygenase (*CDO1*, Genbank accession AY804144) and noted significant expression in the mold morphotype of several *Histoplasma* strains and also determined that the predicted protein would be over double the 10,500 dal reported by Kumar et al. Our report demonstrates (in the class 1 Downs strain, the class 2 G271B strain and two Panamanian strains, 184AS and 186AS) that the *CDO1* gene is expressed in both the mold and yeast morphotypes and both morphotypes show significant CDO activity. Furthermore, we show via a FLAG-tag analysis that the expressed protein is approximately 24.7 ± 2.4 kd, in agreement with the putative protein sequence (determined from cDNA sequence) which yields 23.8 kd and is consistent with most other eukaryotic CDO enzymes. Additionally, we demonstrate that intracellular cysteine levels are actually significantly higher in the mold form of the two Panamanian strains, 184AS and 186AS, equal in both mold and yeast in the class 1 Downs strain and significantly higher in yeast of the more pathogenic class 2 G217B strain.

## 1. Introduction

*Histoplasma capsulatum* (*Hc*) is a dimorphic pathogenic fungus that causes histoplasmosis, a common respiratory disease in humans. It is estimated that approximately 500,000 people are infected each year in the United States alone [1]—the worldwide incidence, however, must be far greater [2]. *Hc* grows in soil in a multicellular mold morphotype but converts to a single-cell yeast morphotype within the host. This dimorphic shift can be reproduced in the laboratory via changing incubation temperature from 25 °C (mold) to 37 °C (yeast). This mold-to-yeast conversion is required for progression of the disease [3]. Therefore, researchers have sought to understand the molecular biology of *Hc* dimorphism. In the late 20th century, it was discovered that sulfhydryl compounds, particularly the amino acid cysteine, appeared to play a critical role in *Hc* dimorphism [4,5]. A major hypothesis is that cysteine, in some fashion, plays a role in intracellular redox which either acts as part of a signal cascade to induce the shift into yeast and/or is needed for maintenance of the yeast morphotype [6]. Maresca et al., in 1981 [4], reported that a yeast-specific cytosolic cysteine oxidase (CDO; cysteine dioxygenase EC 1.12.11.20) appears early in the mold-to-yeast shift (3–4 days after the 25 to 37 °C shift) and hypothesized that this enzyme is required for dimorphism (i.e., conversion of the mold morphotype into the yeast morphotype). Kumar et al. [7] reported the purification of *Hc* CDO and determined the enzyme to have a molecular weight of 10,500 ± 1,500 daltons and to be highly specific for l-cysteine (converting it, with O_2_, to cysteinesulfinic acid; CSA) and reported that it is only present in the yeast morphotype. Therefore, a common assertation in the *Hc* literature is that *Hc* CDO is yeast specific, is approximately 10,500 daltons and plays an important role in dimorphism. Experiments in our lab over the past few years, however, do not coincide with this literature. Hence, the primary aim of this report is to further explore expression of the *CDO1* gene (previously identified in our lab; GenBank accession AY804144), CDO enzyme activity and cysteine levels in four strains of *Hc*. We report here that *CDO1* expression and CDO activity is not yeast specific as there is also significant expression and activity in the mold morphotype. FLAG-tag expression analysis showed the enzyme is over double the previously reported size 10.5 kd of Kumar et al. [7] at approximately 24.7 ± 2.4 kd. This value is in agreement with the putative amino acid sequence, determined from the cDNA, which yields 23.8 kd. Additionally, in contrast to expectations within the literature, intracellular cysteine levels are generally equal to or higher in the mold morphotype than the yeast morphotype.

## 2. Materials and Methods

### 2.1. Strains and Growth

*Histoplasma capsulatum* Downs strain (class 1; North American clade 1; NAm 1) was obtained from ATCC (38904); strains G271B (class 2; NAm 2) and Panama strains (class 3) G184AS and G186AS, were kind gifts from Bill Goldman at the University of North Carolina. Strain designations and clade assignments are further described by Keath et al. [8], Kasuga et al., [9], Vincent et al., [10,11] and Teixeira et al. [12]. Cells were grown to mid log phase in GYE medium (2% *w*/*v* glucose, 1% *w*/*v* Bacto yeast extract) to replicate the experiments of Kumar et al., or in HMM defined medium [13]. The yeast morphotype was grown with gyratory shaking at ~150 rev/min at 37 °C. The mold morphotype was grown with gyratory shaking at 100 rev/min at 25 °C. Yeast were harvested by centrifugation at 1000× *g* for 5 min. Mold cells were harvested by filtration onto a 0.45 um Millipore filter as described previously [14].

### 2.2. DNA and RNA Extraction and Blotting

DNA and RNA were extracted from actively growing cells as described previously [14]. Briefly, cells were harvested by filtration (mold) or centrifugation (yeast) and suspended in RNA buffer (0.1M sodium acetate, pH 5.0, 0.2M NaCl, 0.2% w/v sodium dodecyl sulfate) or DNA buffer (0.1M Tris pH 8.0, 0.1M EDTA, 0.25M NaCl) along with 0.5 mm glass beads and phenol:chloroform (5:1). The tubes were agitated until approximately 80% of the cells were broken as determined by microscopic examination. The nucleic acid was precipitated from the aqueous fraction by adding 2 volumes of ethanol and stored at −20 °C. Northern blotting was performed as previously described [14].

### 2.3. Quantitative PCR

Primer pairs for *CDO1* were designed to yield a product of approximately 112 bp (5′-3′; CDO1F1, GGGCTTGCATAAAATCTCCA and CDO1R1, CCGTCTTCTCGTCAAAGAGG) were validated (R^2^ = 0.99; efficiency = 93%). Total RNA was treated with RQ1 RNase-free DNase (Promega) at 37 °C for 30 min according to the manufacturer’s directions to eliminate contaminating DNA. The RNA was then extracted using phenol:chloroform (5:1) pH = 4.5 and precipitated by adding 1 volume 3M sodium acetate and 2 volumes of ethanol. The precipitated RNA was harvested by centrifugation and dissolved in sterile water. The RNA was used to create cDNA with the RETROscript kit (Ambion) with random decamers according to the manufacturer’s directions. As an internal control, 18s primers and competimer mix (2:8) from Quantum RNA 18s kit (Thermo Fisher Scientific) were used. No-template controls and no-reverse transcriptase controls were also included. For PCR, each 20 µL reaction contained 10 µL of Full Velocity Sybr Green QPCR mix (Stratagene), 0.2 µL of cDNA, 0.3 µL of 1:500 diluted ROX reference dye (Stratagene), and a final primer concentration of 50 nM of each primer. Reverse transcriptase was added to a standard reaction (+RT) and minus RT reactions had no reverse transcriptase. Each of the PCR reactions was done in triplicate on a Mx3000p real-time thermocycler (Stratagene) as follows: 95 °C, 10 min denature; 40 cycles of 95 °C 20 s, 60 °C 30 s, 72 °C 30 s. All real-time quantitative PCR was analyzed with the Mx3000p real-time PCR analysis software (Stratagene). Assays were performed with triplicate independent RNA preparations.

### 2.4. Standard and Fusion PCR

The standard PCR reactions were set up with a volume of 25 or 50 µL with 1–10 ng DNA template, 1× Advantage 2 polymerase (Takara Bio) buffer, 0.2 mM each dNTP, 0.2 µM each of forward and reverse primers and 1× Advantage 2 polymerase. Typical cycling parameters were 30 cycles of the following: denature at 95 °C for 10 s, anneal at 55–68 °C for 30 s, and extend at 68–72 °C for 1 min/kb of DNA fragment. Fusion PCR of DNA fragments was performed by the method of Yu et al. [15], which uses overlapping (nested) primers to create desired constructs.

### 2.5. CDO Assay

A 100,000× *g* enzyme extract was prepared according to the method of Kumar et al. [7]. Briefly, cells were harvested by filtration, resuspended in phosphate buffer (pH 7.0) and broken by ballistic disruption with glass beads. Protein content was determined by the Bradford assay (BioRad) according to the manufacturer’s directions. CDO activity was determined by a modification of the method of Stipanik et al. [16]. Assay buffer (5 mM cysteine-HCl, 2 mM NAD, 5 mM Hydroxylamine HCl, and 0.25 mM (NH_4_)_2_Fe(SO_4_)_2_ made in 100 mM PO_4_ buffer) was prepared fresh for each assay. A standard curve of cysteine sulfinic acid (CSA, Sigma Aldrich) was made from 5 to 0.005 mM. A volume of 100 µL of assay buffer was added to 100 µL of the clarified lysate and incubated for 30 min at 37 °C. An equal volume of 10% (*w*/*v*) trichloroacetic acid (TCA) was added to stop the reaction. Control reactions had TCA added immediately before the enzyme was added. The solution was centrifuged at 10,000× *g* for 5 min to precipitate protein. 200 µL of the supernatant was placed on a Dowex minicolumn (Sigma Aldrich, approximately 0.3 mL bed volume) and eluted with water. CSA production was measured by the ninhydrin reaction as follows: 100 µL of the eluate was added to 50 µL of ninhydrin reagent (Sigma Aldrich). The mixture was then incubated at 90 °C for 10 min. After cooling to room temperature, 150 µL of 95% ethanol was added and mixed and the absorbance at 550 nm was measured. These reactions were linear and directly proportional to enzyme added. To ensure that the assay was, in fact, measuring CSA produced, reactions were also analyzed via thin layer chromatography on PEI-cellulose plates (Sigma-Aldrich) and compared to authentic CSA. Ascending chromatography was done by using butanol-acetic acid-water (12:3:5) as the solvent. A standard of 10 to 10 nM of cysteine and CSA was spotted along with samples. After approximately 30 min of solvent flow, the chromatogram was developed by spraying with Ninhydrin Spray (Sigma) according to the manufacturer’s directions.

### 2.6. CDO Flag-Tag Analysis

Genomic DNA of the *CDO1* gene with 1.5 kb of its native promoter sequence was used to create Flag-tag fusions. Fusion PCR was used to attach the sequence for the 8 amino acid FLAG-tag [17] immediately after (in frame) the putative ATG start codon or immediately before (in frame) the putative TGA stop codon to yield N-terminal and C-terminal fusions of the *CDO1* gene to the C-terminus and the N-terminus of *CDO1,* in separate constructs, which were sequenced to confirm proper placement of the FLAG-TAG. The products were cloned in the *Histoplasma* telomere vector, pRPUI [18] which contains a *Ura5* marker for selection. *Hc* strain 186AS^Ura5-^ was electroporated [18] with the plasmid. pRPUI without the CDO FLAG-TAG fusion was used as a negative control. Protein from transformants was extracted from mid-log phase cells as follows: cells were washed in ice cold Dulbecco’s PBS three times and then an equal volume of 0.5 mm glass beads was added. Cells were broken via ballistic disruption by vortexing on maximum speed on a Vortex Genie (Scientific Industries) for three cycles of 1 min followed by 1 min of cooling in crushed ice. The cells were then centrifuged at 10,000× *g* for 15 min at 4 °C. The clarified lysate (approximately 20 µg protein) was mixed with 10× loading buffer to yield a final concentration of 0.125 M Tris-HCL buffer (pH 6.8) containing 2% *w*/*v* SDS, 10% *v*/*v* glycerol, 5% *v*/*v* 2-mercaptoethanol, and 0.025% *w*/*v* bromophenol blue. The protein was separated on a 15% *w*/*v* polyacrylamide gel and then blotted onto nitrocellulose membrane. The transfer buffer was 25 mM Tris HCL, 192 mM glycine, and methanol (20% *v*/*v*, pH 8.3). Free binding sites of the membrane were blocked with the incubation of 5% *w*/*v* commercial nonfat dry milk in phosphate buffered saline (PBS), 0.3% Tween 20 (pH = 7.5). The membrane was then probed with an alkaline phosphatase linked Anti-FLAG antibody (Sigma) according to the manufacturer’s directions.

### 2.7. Intracellular Cysteine Analysis

To measure the total intracellular cysteine we subjected mid-log phase cells to sulfosalicylic acid extraction by the method of Dominy et al. [19]. Briefly, cells were extracted with sulfosalicylic acid followed by reduction with dithiothreitol in 10 N NaOH. The reduced samples were acidified with glacial acetic acid and reacted with ninhydrin followed by absorbance analysis at 560 nm.

## 3. Results

### 3.1. CDO1 Expression

**Figure 1:**
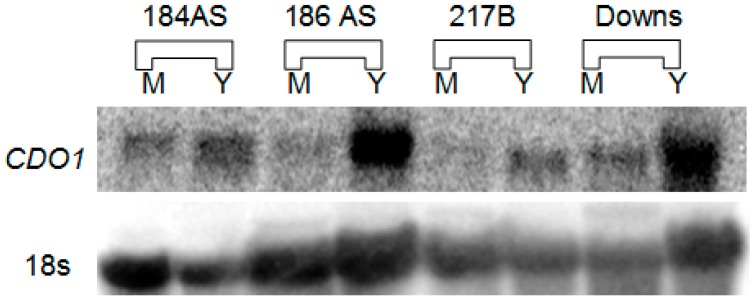
Northern blot analysis of *CDO1* (top) probed with *Hc* full length ^32^P-*CDO1* compared to 18s RNA: competimer control (bottom) stripped then probed with 18s ^32^P-rDNA. M, mold morphotype; Y, yeast morphotype.

**Figure 2:**
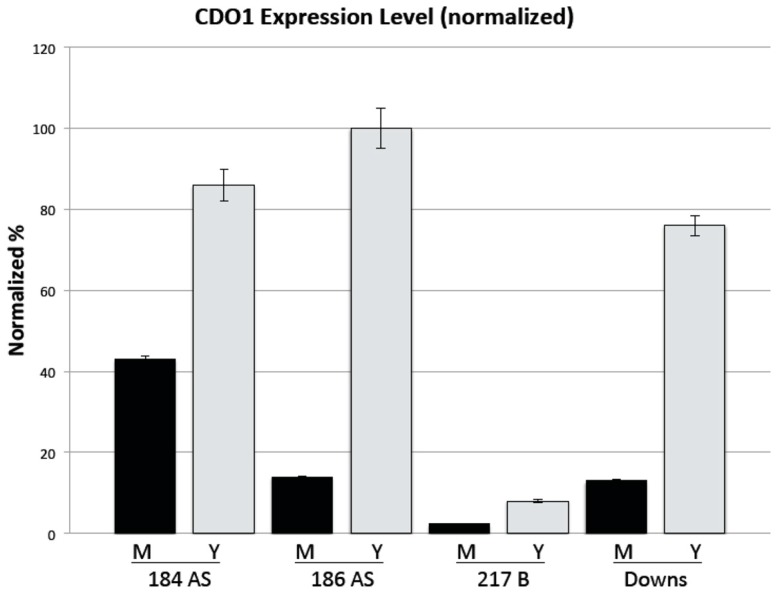
*CDO1* expression determined by quantitative PCR. M, Mold; Y, Yeast. Expression was normalized to 186AS yeast = 100%. Error bars are the standard error of the mean of triplicate independent experiments.

Figure 1 shows a northern blot of total RNA probed with a full-length ^32^P radiolabeled Hc CDO1 probe. A single transcript of approximately 1.3 kb (compared to RNA standards) was detected in each strain. This qualitative assay demonstrates that the CDO1 transcript is more abundant in yeast than in mold, but it is expressed at significant levels in the mold morphotype also. Only a single transcript, of approximately the same size, was detected in each lane. Figure 2 shows a quantitative determination of CDO1 expression via real-time quantitative PCR. Although the yeast morphotype demonstrated greater expression, significant expression of CDO1 was likewise seen in the mold morphotype of all four strains.

### 3.2. CDO Enzyme Activity

**Figure 3:**
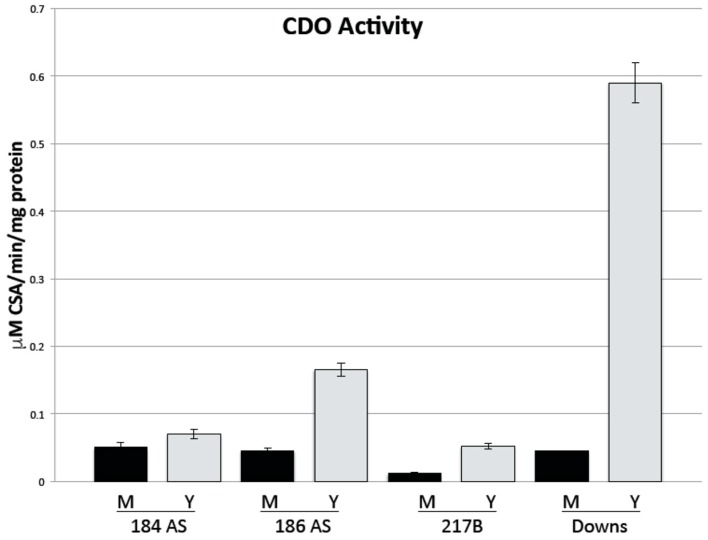
Cysteine dioxygenase activity. M, mold; Y, yeast. Activity was normalized to protein level within the extract. Error bars are the standard error of the mean of triplicate independent experiments.

CDO enzyme activity was determined by measurement of CSA production. CDO activity was more abundant in the yeast morphotype but significant activity was also noted in the mold morphotype (Figure 3). The Downs yeast had remarkably greater activity than any other strain.

### 3.3. CDO Molecular Weight

**Figure 4:**
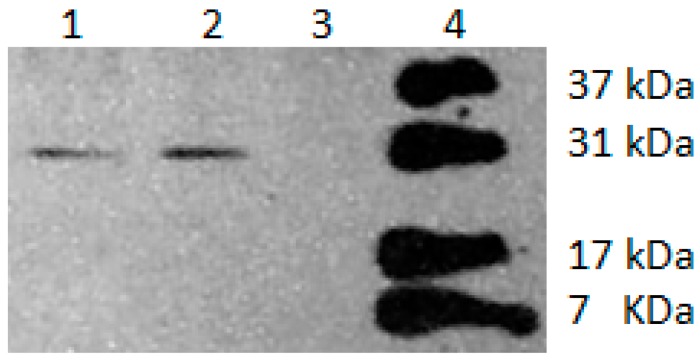
Western blot of FLAG-tagged Hc CDO probed with anti-FLAG antibody. Lane 1, C-terminus fusion; Lane 2, N-terminus fusion; Lane 3, no FLAG-tag control; Lane 4, protein MW standard. Size estimate determined by analysis of three independent blots = 25.7 kDa. Standard deviation was ± 2.4 kDa.

CDO enzyme was determined to have a molecular weight of 25.7 ± 2.4 kd by comparison of both an N-terminal Flag-tag construct and a C-terminal tagged construct as shown in Figure 4. The 8 amino acid FLAG-tag would contribute approximately 1 kd to this value, and thus the final estimated Hc CDO enzyme size is 24.7 kd.

### 3.4. Intracellular Cysteine Levels

**Figure 5:**
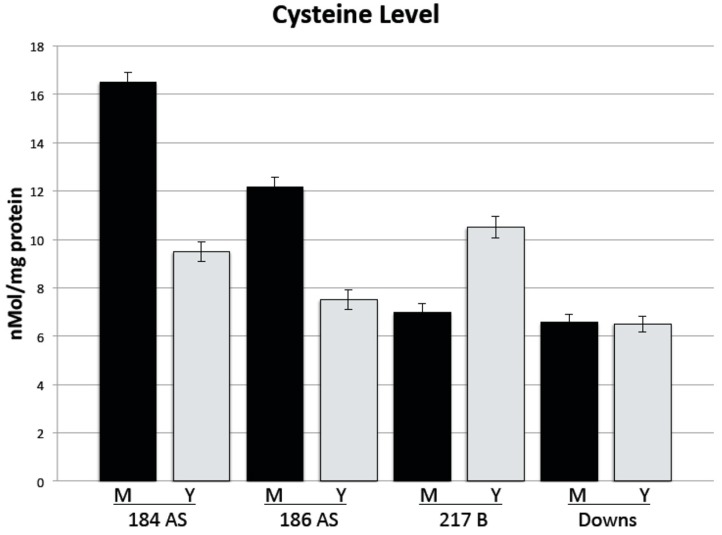
Intracellular cysteine level. M, mold; Y, yeast. Cysteine levels were normalized to protein level. Error bars are the standard error of the mean of triplicate independent experiments.

Intracellular cysteine levels (Figure 5) were significantly higher in the mold versus the yeast of the two Panamanian strains (184AS and 186AS). In strain G217B, however, the mold morphotype had significantly lower cysteine levels (approximately 67%) than the yeast morphotype. In the Downs strain, cysteine levels were essentially the same in both the mold and yeast morphotype.

## 4. Discussion

Since the 1980s, a common assertation within the *Histoplasma* dimorphism literature is that the organism has a yeast-specific cysteine dioxygenase, of approximately 10,500 daltons, which is thought to play an important role in mold-yeast dimorphism. In 2004, however, we isolated and sequenced the *Hc CDO1* gene and cDNA (GenBank AY804144.1). The size of the predicted protein (approx. 24 kd) was over double that reported by Kumar et al. [7]. Additionally, we noted in northern blots that the *CDO1* transcript was also present in mold, albeit in lower amounts than in the yeast. Hence, we sought to investigate *CDO1* expression and activity in four commonly used laboratory strains as well as to determine the size of the expressed protein. Since the Downs strain was used in the original work of Kumar et al., we likewise included this strain for direct comparison.

As noted in Figure 1, *CDO1* was expressed in significant amounts even in the mold morphotype of all four strains. Quantitative real-time PCR (Figure 2) demonstrated that the mold morphotypes of all four strains also express *CDO1*. Interestingly, the most pathogenic strain tested, G271B, had dramatically less expression than the other, less pathogenic, strains. In strain G217B, the mold morphotype expressed only approximately 1/3 as much *CDO1* as the yeast (Y/M = 3.3). This difference in Y vs. M in G217B is consistent with the ratio determined by Edwards et al. for the same strain [20] (approx. Y/M = 2.4; https://microbiology.osu.edu/RappleyeHistoplasma). Interestingly, Hwang et al., [21] reported a *CDO1* Y/M ratio of 11 in the same G217B strain via genomic shotgun microarray. *CDO1* expression in our study was weakest overall in strain G217B while the other three strains had vastly greater expression. Approximate ratios (Y/M) for the other three strains were 184AS, 2.0; 186AS, 7.14; Downs, 5.8.

Since it is possible that transcript levels may not equate with enzyme activity, we measured CDO activity (Figure 3). Again, the mold morphotype of all four strains showed significant CDO activity albeit not as much as the yeast morphotype. The most striking observation was the dramatic difference in Y versus M in the Downs strain—the yeast morphotype had approximately 13-fold greater activity in yeast than in the mold. The other Y/M ratios were 184AS, 1.4; 186AS, 3.6; G217B, 4.3. It is interesting that the strain examined by Kumar et al., [7] was the Downs strain and this is the strain with the greatest CDO activity.

Kumar et al., [7] established the molecular weight of the purified CDO enzyme as approximately 10,500 daltons—surprisingly small relative to other known CDO enzymes. Since our northern blot data (Figure 1) showed that all four strains had *CDO1* transcripts of similar size and this is consistent with our 1.2 kb cDNA sequence (GenBank AY804144.1), we selected the 186AS *CDO1* gene to create FLAG-tag fusions with the 8 amino acid FLAG sequence [17] on either the N-terminus (immediately after, and in frame, of the putative ATG start codon) or C-terminus (immediately before, and in frame, of the putative TGA stop codon) in a uracil auxotroph of the same strain. As shown in Figure 3, identical size bands for both the N-terminal fusion and the C-terminal fusion were seen on western blots probed with anti-FLAG antibodies. Comparison to molecular weight standards yielded an estimated size of 25.7 ± 2.4 kd. Once the approximately 1 kd contributed by the 8 amino acid FLAG-tag is subtracted, the final estimate is approximately 24.7 kd (22.3 to 27.1 kd). The calculated molecular weight based on the predicted amino acid sequence (GenBank AY804144.1) is 23.8 kd, which is well consistent with the determination via western blotting.

The reason the much lower 10.5 kd estimate was obtained by Kumar et al. is unknown. The protein was purified by a cysteine-Sepharose affinity column and then labeled with ^125^I to enable detection of the small amounts of protein present. The molecular weight was then determined by SDS gel electrophoresis as compared to protein molecular weight markers. The researchers commented on the unusually small size as compared to other known CDO enzymes from eukaryotes. They noted that rat liver CDO, for example, was approximately double in size and proposed that iodination may have affected the apparent molecular weight on the SDS gel. However, they ultimately rejected this hypothesis since a 2D gel analysis of both iodinated and non-iodinated protein demonstrated that both the iodinated and non-iodinated spot were at the same position on the SDS gel. What then might account for the low size estimate? Since mobility in SDS gel electrophoresis depends strongly on proportional binding of SDS micelles, aberrant binding will create errors in molecular weight estimates. Rath et al., for example, notes that variances of -10% to +30% are well known [22]. These variances, however, do not seem to able to account for a gel shift as dramatic as 23.8 kd to 10.5 kd. Perhaps the 10.5 kd protein results from some kind of cleavage during the purification process. In any event, the 23.8 kd CDO estimate described in this report (Figure 4) is consistent with both the cDNA sequence data and predicted protein size as well as the size of FLAG-tag constructs and the typical molecular weights of most eukaryotic CDO enzymes which are generally approximately 23–24 kd, e.g., rat (GenBank BAA11925), human (BAA12872), mouse (AAK53364), *Aspergillus* (GCB23511), *Phytophtora* (KUF76318), *Talaromyces* (OKL60606), etc.

The intracellular cysteine levels are also intriguing (Figure 5). Generally, high cysteine (or other sulfhydryls) in growth media are correlated in the literature with cells growing in the yeast morphotype—perhaps via providing a more reducing environment. Rippon [23], for example, demonstrated that electrolytically lowering the redox of the growth media would allow the mold-to-yeast shift to occur at the “non-permissive” (for the yeast) temperature of 25 °C provided the medium redox was lowered to +46 mV. Hence, it is postulated that this sulfhydryl compound, in some manner, alters intracellular redox and thereby, in some manner, affects the signal cascade and/or the maintenance of the yeast morphotype. Thus, it seems somewhat counterintuitive that the cysteine levels in the two Panamanian strains (184AS and 186AS) were significantly higher in the mold than the yeast (Figure 4). The Downs strain had equal levels in both morphotypes and only the more pathogenic G217B strain demonstrated significantly higher cysteine levels in the yeast.

Clearly the role of CDO and cysteine itself in *Hc* dimorphism is complex. In future, it would be useful to create either *CDO1* knockout or RNAi knockdown strains. Our numerous attempts to create a *CDO1* knockout have been unsuccessful. Perhaps this is because the knockout is lethal in *Hc*. However, the gene has been shown to be non-essential in several organisms. In mice, *CDO1* -/- are viable even without taurine supplementation. In *Candida albicans*, deletion of the *CDO1* homolog, *CDG1*, resulted in reduced hypha formation (in the presence of cysteine) and showed reduced virulence in a mouse model of disseminated infection [24]. However, given the challenge with allelic replacement methods in *Hc*, it simply may be that more attempts are required.

We note in this report that there are remarkable differences in the class 1 vs. class 2 strains: Downs yeast had the highest expression of *CDO1* as compared to G217B which had the lowest expression (Figure 2), and Downs yeast had the greatest CDO enzyme activity by far and G217B the least (Figure 3). It is curious, however, that Medoff et al. [3] reported that the class 1 strains (Downs) require cysteine to progress through the mold-to-yeast transition but class 2 strains (e.g., G217B) do not. Perhaps the Downs strain, and likely other class 1 strains, have a different carbon flow in the cysteine-associated pathway and/or different sulfur assimilation pathways—this would be an interesting investigation for the future.
